# Study of adherence to exercise in heart failure: the HEART camp trial protocol

**DOI:** 10.1186/1471-2261-14-172

**Published:** 2014-11-29

**Authors:** Bunny J Pozehl, Kathleen Duncan, Melody Hertzog, Rita McGuire, Joseph F Norman, Nancy T Artinian, Steven J Keteyian

**Affiliations:** University of Nebraska Medical Center, College of Nursing, 1230 O Street, Suite 131, Lincoln, NE USA; Division of Physical Therapy Education, University of Nebraska Medical Center, Omaha, NE USA; Wayne State University, College of Nursing, Detroit, MI USA; Department of Medicine, Henry Ford Hospital, Detroit, MI USA

**Keywords:** Heart failure, Exercise adherence, Moderate intensity exercise, HEART camp, Heart failure society of America guidelines

## Abstract

**Background:**

Adherence to the Heart Failure Society of America (HFSA) 2010 guidelines recommending 30 minutes of supervised moderate intensity exercise five days per week is difficult for patients with heart failure (HF). Innovative programs are needed to assist HF patients to adhere to long-term exercise. The objective of this prospective randomized two-group repeated measures experimental design is to determine the efficacy of a behavioral exercise training intervention on long-term adherence to exercise at 18 months in patients with heart failure.

**Methods/Design:**

A sample size of 246 subjects with heart failure will be recruited over a 3 year period. All subjects receive a cardiopulmonary exercise test and 9 supervised exercise training sessions during a 3 week run-in period prior to randomization. Subjects completing at least 6 of 9 training sessions are randomized to the HEART Camp Intervention group (HC) or to a standard care (SC) exercise group. The HC intervention group receives cognitive-behavioral strategies that address the intervention components of knowledge, attitudes, self-efficacy, behavioral self-management skills and social support. The SC group is provided access to the exercise facility and regular facility staff for the 18 month study period. The primary aim is to evaluate the effect of HEART Camp on adherence to exercise, with our central hypothesis that the HC group will have significantly better adherence to exercise at 18 months. Secondary aims include evaluating which components of the HEART Camp intervention mediate the effects of the intervention on adherence; evaluating the effect of HEART Camp on specific health outcomes; exploring selected demographic variables (race, gender, age) as potential moderators of the effect of the HEART Camp intervention on adherence; and exploring the perceptions and experiences that contextualize exercise adherence.

**Discussion:**

The HEART Camp intervention is the first to test a multi-component intervention designed to improve long-term adherence to exercise behavior in patients with HF. Improving long-term adherence to exercise is the logical first step to ensure the required dose of exercise that is necessary to realize beneficial health outcomes and reduce costs in this burdensome chronic illness.

**Trial registration:**

Clincaltrials.gov NCT01658670.

## Background

The guidelines from Heart Failure Society of America [1] recommend 30 minutes of moderate intensity exercise in a supervised setting five days per week, but this is difficult for patients with heart failure (HF). Studies indicate that between 40% and 91% of patients with heart failure do not engage in any regular exercise [2–5]. A majority (61%) of patients with heart failure report that adherence to an exercise regimen is more difficult than any of the other required behavioral changes for heart failure including diet modification, medication adherence, smoking cessation, or keeping appointments [6, 7]. Patients identify lack of motivation [5, 6, 8, 9], lack of energy [5, 6, 8], and the presence of physical symptoms as the primary reasons for non-adherence to exercise [5, 6, 8–10]. Patients report reluctance to initiate exercise due to a lack of skills for exercise [11] and a fear of increasing physical activity with a “bad heart” [12, 13]. Despite this identified need, specific programs to teach heart failure patients how to exercise and adhere to exercise behavior over time do not exist [11]. Complicating this further has been the lack of reimbursement for patients to participate in supervised programs where they might learn how to exercise and maintain the behavior over time. Even with the Centers for Medicare and Medicaid (CMS) now covering cardiac rehabilitation for beneficiaries with heart failure due to reduced ejection fraction, statistics show that patients do not continue with adherence to exercise once the rehabilitation is completed [14, 15]. Clearly, innovative programs are needed to assist heart failure patients to gain the knowledge, skills and motivation for long-term adherence to exercise behavior.

While dozens of single-site trials have described the effects of exercise on pathophysiology and exercise intolerance in heart failure, few if any of these trials report or critically examine adherence data. Often these studies just report results for those patients who completed the requisite amount of exercise versus all patients enrolled in the trial. Studies that focus on adherence to exercise are lacking and as a result, we know very little about what it takes to adhere to exercise when living with chronic heart failure. Adherence to exercise in heart failure is problematic as the six-month dropout rates from exercise studies are as high as 40% [16]. Prior to the 2010 guideline recommending five sessions per week, most exercise training studies aimed to achieve three sessions per week; even with this more modest goal, patients on average completed only 1.7 sessions weekly at the end of 12 months [17]. The only study to examine adherence to exercise beyond 12 months in patients with heart failure is the multi-center HF-Action trial (Heart Failure – A Controlled Trial Investigating Outcomes of Exercise Training) that reported adherence as low as 34% at the end of 24 months. Current evidence suggests that exercise has a dose–response effect in heart failure [18] and that when patients do not adhere to exercise; the realized benefits in peak VO2 and quality of life are significantly less [19, 20] and not maintained [20, 21]. Thus, there is a critical need for a paradigm shift to focus on adherence to exercise in this chronically ill heart failure population. This study uses behavioral strategies previously tested in other populations and in the preliminary work of these investigators to improve adherence to exercise (quantitatively) and to understand the process of adherence to exercise (qualitatively) in patients with heart failure.

This paper presents the study protocol for a prospective randomized controlled trial that is currently in progress. The Heart failure Exercise And Resistance Training Camp (HEART Camp - HC) intervention group is compared to a SC exercise group. The central hypothesis is that the HC group will have significantly better adherence to exercise at 18 months than the SC group. Those in the HC group receive a multicomponent behavioral intervention to assist them in adhering to exercise. The intervention components of knowledge, attitudes, self-efficacy, behavioral self-management skills and social support are measured and will be tested as possible mediators of the effect of the intervention on adherence. The intervention is being carried out in two sites in the United States (i.e., Detroit, MI with a predominately African-American population and Lincoln, NE with a primarily Caucasian population of patients). Race, gender and age will be explored as potential moderators of the effect of the HEART Camp intervention on adherence. Finally a qualitative component will explore the perceptions and experiences that contextualize exercise adherence.

## Methods/Design

### Ethics approval

This study was approved by Institutional Review Boards at the University of Nebraska Medical Center in Omaha and at Wayne State University and Henry Ford Health System in Detroit. Written informed consent is obtained per protocol from all participants prior to enrollment in the study.

### Study aims

- Aim 1. To evaluate the effect of HEART Camp on adherence to exercise. We measure adherence using self-reported exercise validated by objective heart rate monitor data. Our *working hypothesis* is that subjects in the HC intervention group will have better adherence to exercise than the SC group over time (6, 12 and 18 months) with a maximum difference expected at 18 months.
- Aim 2. To evaluate which components of the HEART Camp intervention mediate the effects of the intervention on adherence. Our *working hypothesis* is that participation in the HC intervention will improve the targeted components (greater knowledge, better attitudes, greater self-efficacy for exercise, more use of self-management skills, and higher perceived social support) resulting in increased adherence to exercise over time (6, 12 and 18 months).
- Aim 3. To evaluate the effect of HEART CAMP on specific health outcomes. We measure physical function using the 6 minute walk test; psychological function using the PROMIS - 29 Profile v.1; symptoms using the Dyspnea Fatigue Index; and quality of life using the Kansas City Cardiomyopathy Questionnaire. Our *working hypothesis* is that subjects in the HC group compared to the SC will have higher levels of physical function, psychological function and quality of life and lower levels of symptoms over time (6, 12 and 18 months).
- Aim 4. To explore selected demographic variables (race, gender, age) as potential moderators of the effect of the HEART Camp intervention on adherence.
- Aim 5. To explore the perceptions and experiences that contextualize exercise adherence. Thirty minute open-ended interviews will be used to explore perceived barriers, challenges, reinforcers/support mechanisms among study participants, individual coaches, and group session leaders during the adoption, transition, and maintenance phases of the HC intervention.

### Research design

This study uses a prospective randomized two-group repeated measures experimental design with four data collection points (baseline, 6, 12 and 18 months). This study also uses qualitative methods within the randomized control trial design to assess the context for exercise adherence in the intervention group [22–24]. Both the HC group and the SC group receive paid access (free membership) to an exercise facility over the 18 month study period and both groups receive usual care for heart failure from the participating institutions.

### Sample size

The required sample size for the study was estimated to compare groups on the primary endpoint of adherence at 18 months. In the calculations, the level of adherence in the control group was set at 0.25, the level of crossover seen in HF-Action during the first year. Reported adherence in the HF-Action exercise group was 0.38 at 12 months and 0.36 at 18 months. Having only two sites in the current study should allow greater control over study implementation, which should increase adherence. We expect to achieve adherence of at least 0.50 in the HC intervention group based on an adherence level of 86% in our R15 trial during the three months after the active intervention phase of that trial. A one-tailed z-test of the difference in proportions of this magnitude would have power of 0.90 (α = 0.05) with a total sample size of 126 participants.

### Subjects

We will recruit 246 patients (123 participants from each study site). Assuming that 10% might not meet the medical criteria for safe participation in the study and that 30% will fail to attend at least 6 of the 9 planned sessions during the run-in period, 148 subjects will be randomized. Using HF-Action’s 12-month dropout rate of 32% as a guide, an additional 15% attrition is assumed over the 18 months of the study, resulting in the final desired sample size of 63 per group (total N = 126) at 18 months.

### Recruitment and enrollment

Two sites were chosen for their ability to recruit and enroll participants from a diverse geographic area: BryanLGH Heart Institute in Lincoln, NE and The Henry Ford Health System in Detroit, MI. The Lincoln site is a cardiology practice with 15 cardiologists. This site was used in our previous work and allowed us to successfully recruit participants in a pilot R15 study. Of the 42 participants included in that study, only one person was African American. The Henry Ford Health System with 82% African-American patients, was chosen for the second site specifically because of its access to underserved and ethnic minorities and its previous experience enrolling patients with HF in exercise trials. Combined, the two enrollment sites served 3,563 patients with HF in 2010 when this study was conceptualized.

### Participants

Eligibility according to inclusion and exclusion criteria is summarized in Table 1. Cardiology providers and a co-investigator with ethical access at each study site identify potential subjects and screen for eligibility.

**Table 1:** **Inclusion and exclusion criteria for the HEART camp study**

Inclusion criteria	Exclusion criteria
• Diagnosis of heart failure	• Clinical evidence of decompensated HF
Stage C chronic HF	• Unstable angina pectoris
Confirmed by echocardiography and clinical evaluation)	• Myocardial infarction, coronary artery bypass surgery, or biventricular pacemaker less than 6 weeks prior
Either Heart Failure preserved Ejection Fraction (HFpEF) or Heart Failure reduced Ejection Fraction (HFrEF)	
• Nineteen years of age or greater	• Orthopedic or neuromuscular disorders preventing participation in aerobic exercise and strength/resistance training
• Able to speak and read English	• Participation in 3 times per week aerobic exercise during the previous 8 weeks.
• Telephone access in home (land line or cell phone)	• Cardiopulmonary stress test results that preclude safe exercise training
• Stable pharmacologic therapy per guidelines for past 30 days (i.e., stable doses of beta-blocker, ACEI or ARB, diuretic)	• Plans to move more than 50 miles from the exercise site within the next year
	• MVO2 in females >21 ml/kg/min and in males >24 ml/kg/min
	• Pregnancy - If participant is pregnant or plans to become pregnant during the study

### Baseline exercise testing and training

Per the 2010 HFSA guideline recommendation, all participants receive a cardiopulmonary stress test prior to exercise [1]. Once participants have consented to participate in the study, trained staff at the respective sites conduct the cardiopulmonary stress test (CPX). The 2010 HFSA guidelines recommend moderate intensity exercise training in a supervised setting therefore both groups receive 9 exercise training sessions during a 3 week run-in period and continue to exercise after randomization in the supervised setting of a health care exercise facility. Nine exercise training sessions prior to randomization provides training in moderate-intensity aerobic exercise (40%-80% Heart Rate Reserve; HRR) and provides the supervision we feel is necessary to assure the safety of all participants for exercise [5, 25–28]. If a participant is deemed unsafe/unable to exercise at the completion of the run-in period, he or she is not randomized to participate in the study. In addition to assuring safety, the decision to only randomize individuals who attend at least 6 of the 9 sessions (minimum of 2 sessions for each of the 3 weeks of run-in) was made to provide an adequate intent-to-treat analysis when examining adherence. We recognize this may limit generalizability, but we feel it is an important first step to ensure safety when investigating adherence to exercise in individuals who have an initial desire to exercise and an informed understanding of exercise expectations.

### Intervention

#### Conceptual framework/background

The HC intervention is based on cognitive-behavioral strategies that were effective in our pilot work and are supported in the literature. Effective strategies that have been used to change physical activity behavior in the general population include goal setting [29–34], self-monitoring [31, 32, 34–38], frequent and prolonged contact [31, 34, 39–41], feedback and reinforcement [29, 30, 34, 41, 42], self-efficacy enhancement [30, 31, 33–35, 38, 42, 43], modeling [31, 44], problem solving and relapse prevention [29, 32, 37, 45, 46]. We use a multi-component approach of group-based and individual-based intervention delivery, reported to be successful in changing physical activity behavior [34]. The intervention includes fitness testing, exercise prescriptions, supervised exercise, goal setting, monitoring and relapse prevention, all of which have been shown effective in increasing physical activity in a recent meta-analysis of interventions to increase physical activity in healthy adults [47]. Interestingly, this meta-analysis showed that studies based solely on social-cognitive or transtheoretical theory had smaller effect sizes, while behavioral interventions had larger effect sizes [47]. It is our belief that social-cognitive aspects of knowledge and self-efficacy may be more important for the patient with HF than for healthy adults because HF patients report lack of knowledge, skills and confidence needed for exercise [11–13]. The model of future-oriented motivation and self-regulation proposed by Miller and Brickman [48] has also contributed to the conceptualization of this study. The Miller and Brickman model synthesizes aspects of Bandura’s social-cognitive theory (focused on short-term or proximal goals) and adds theory to achieve future goals and long-term behavior change. Addressing future or long-term goals is consistent with the mission of the NIH Health Maintenance Consortium (HMC) which studies long-term behavior change [49]. The HMC emphasizes a need to understand the surrounding context that can either facilitate or impede the behavior change process and to focus on the mechanisms of change that include: knowledge, self-efficacy, behavioral skill-building and social reinforcement [49, 50]. Because the positive effects from behavior change interventions tend to diminish over time without continued support, the mechanisms of change in the HC intervention are continued throughout the 18 month study period [50].

#### Intervention components

Multi-component group-based and individual-based intervention delivery are included as they have been the most successful approaches in changing physical activity behavior [34]. Specific quantitative measures for each of the components of the HC intervention (i.e., knowledge, attitudes, self-efficacy, behavioral self-management skills and social support) are included in order to evaluate which components mediate the effects of the intervention on adherence (see Table 2). Furthermore, we are collecting qualitative data from subjects, coaches and group session leaders to better understand the adherence process over time in this patient population. We measure adherence to exercise using self-reported exercise diaries that are validated by heart rate monitor data [51].

**Table 2:** **Intervention components, strategies and delivery**

Intervention component	HEART camp intervention strategies & camp activities	Delivery mode
**KNOWLEDGE**		
Knowledge of exercise training	• Aerobic exercise	9 exercise training sessions delivered by facility staff.
	• Resistance exercise (bands, free weights or circuit equipment)	
	• Heart rate monitor to self-monitor	Weekly meetings with coach for training first 12 months.
	• Rating of perceived exertion (RPE) to self-monitor	
Camp topics on exercise in the daily life of a patient with HF	• HEART Camp Motto – “Choose to Move for Heart Improvement” – Discussion of expectations and feelings related to exercise	Weekly group sessions with the RN (30 minutes of content and 30 minutes of discussion).
	• “Game Day: Firing up your Engine” – Pathophysiology of HF and benefits of exercise	
	• “Telling Your Story” – Sharing symptom experiences and understanding symptoms in relation to exercise	Schedule will repeat every 6 weeks. Subjects expected to attend each session at least once. Subjects encouraged to bring significant others/family to sessions.
	• “Show & Tell” – Bring medications to group session and discuss actions and effects in relation to exercise	
	• “Pack-a-Lunch Day” – Dietary sodium and fluid restrictions (discuss fluid retention and effects on exercise)	
	• “Survival Guide” – How-to exercise safely during life’s ups and downs	
**ATTITUDES**		
Safety (Reducing fear)	• Cardiopulmonary Stress Test (CPX) at beginning of run-in	Facility staff and coach use individual exercise guidelines to adjust exercise
	• Individual exercise guidelines determined from CPX (40%-80% HRR)	
Perceived benefits and barriers	• Complete Barriers Self-Efficacy Scale (BARSE) at each data collection point	Weekly meetings with the coach to discuss barriers, strategies to overcome barriers, and reinforce exercise benefits
	• Weekly discussion with coach to identify individual strategies for overcoming perceived barriers to exercise and reinforcement of benefits	
**SELF-EFFICACY**		
Enactive mastery experiences (performance accomplishment)	• Return demonstrate use of exercise equipment and heart rate monitor	Subject’s weekly meeting with coach
	• Goal setting for weekly exercise & review of weekly graphs of goal accomplishment (total number of sessions, total number of minutes of moderate intensity exercise)	
Vicarious experience	• Sharing successes during group session discussion	Weekly group sessions with RN allow 30 minutes for group discussion and sharing
	• Sharing strategies to overcome barriers during group sessions	
Verbal persuasion	• Education on benefits of exercise	Six weekly group sessions with camp counselor.
	• Web-based educational material related to exercise (HFSA Module on Exercise)	Weekly meetings with coach to discuss goal-setting and feedback.
	• Feedback related to individual progress toward goals	
Physiological & affective States	• Symptom assessment education	Discussed in group sessions and as needed by coach
	• Recognition of warning symptoms to moderate or stop exercise	
	• Symptom management strategies for exercise	
**BEHAVIORAL SELF-MANAGEMENT SKILLS**		
Goal setting	• Goals: number of sessions and number of minutes of moderate intensity exercise per week	Coach reviews with subject each week
Self-monitoring	• Return demonstration of self-monitoring techniques (heart rate, rating of perceived exertion, symptoms)	Facility staff and coach
	• Entry/log in exercise diary	Coach reviews diary weekly
Problem-solving	• Subject notes problems in exercise diary	Coach reviews diary weekly
	• Subject reviews problems with coach	
Barriers management	• Coach reviews BARSE at data collection time points (Baseline, 6 and 12 months)	Coach discusses individual perceptions and strategies
Relapse management	• Phone call to subject if relapse for one week	Coach places phone call to set up face-to-face meeting
**SOCIAL SUPPORT**		
Social relationships	• Six weeks with RN in camp group sessions	Group sessions
	• Weekly interaction with coach during adoption and transition	Weekly coach interaction

#### Intervention phases

The 2009 meta-analysis of interventions aimed to increase physical activity among patients with cardiovascular disease showed large effect sizes in studies that had: (1) a focus on physical activity behavior exclusively, (2) more contact between subjects and interventionists, (3) supervised exercise sessions, (4) fitness testing, (5) face-to-face encounters versus other methods of intervention delivery, and (6) a recommendation for more minutes of activity per week [52]. Findings from these studies and our previous research have guided both the timing and approach for the HC Intervention. We use three phases that gradually decrease the face-to-face interaction with research staff to prepare the participant for self-management of exercise during the last 6 months of the study.

##### Phase 1 – adoption (Baseline-6 months)

The adoption phase emphasizes all components of the intervention: knowledge, attitudes, self-efficacy, self-management skills and social support. Six weekly one-hour group education sessions address creative and fun topics for the patient to learn about exercising with HF in a relaxed and supportive environment. A registered nurse leads the sessions as outlined in Table 2. Sessions occur on a repeating 6-week schedule to allow participants to attend a specific session more than once if they desire. Participants may invite a friend or family member to attend sessions with them. Weekly face-to-face interactions with the coach at the facility focus on attitudes, self-efficacy and behavioral self-management skills, providing participants with continuous social support for exercise. During these meetings, the coach reviews participants’ exercise diaries, facilitates practice of self-management skills (e.g., goal-setting, feedback, problem-solving and discussion of barriers to exercise), addresses the diversity of values and beliefs, and helps adapt the intervention to the culture and life context of participants.

##### Phase 2 – transition (Months 7–12)

Participants are expected to interact with the coach at the facility once a week. If a subject relapses and does not follow through with this weekly meeting, the coach contacts the subject by phone to schedule a face-to-face meeting. Group sessions are available weekly if participants wish to repeat sessions or interact in the group.

##### Phase 3 – maintenance (Months 13–18)

**Figure 1:**
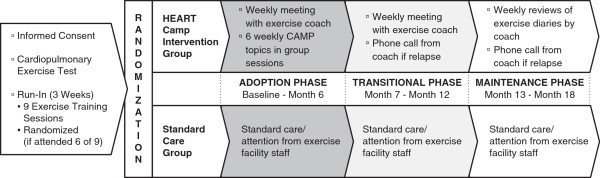
**Study model.**

During the last 6 months, participants are expected to be self-managing exercise and maintaining adherence to 150 minutes of moderate intensity exercise each week. Exercise diaries are submitted weekly, and the coach calls a subject to schedule a face-to-face interaction if relapse occurs during this phase. See Figure 1 for a depiction of the Study Model.

#### Standard care

Given that the HFSA recommends 150 minutes of moderate intensity exercise per week, it would be unethical to ask the control group to abstain from exercise. The SC group is provided access (free membership) to the exercise facility and regular facility staff during the 18 month study period. This membership was provided in order to control for access as a potential confound to adherence since this is noted as a common barrier to exercise adherence [53, 54]. During all three phases of the study, participants in both groups are expected to record all moderate intensity exercise on a weekly diary and wear the heart rate monitor during this exercise. The investigators recognize that the HC intervention group will receive more attention than the SC group; however, more contact between participants and interventionists and face-to-face encounters versus other methods of intervention delivery have been documented to produce larger effects and thus are being evaluated as part of the intervention [47, 52]. Both the HC intervention group and the SC group receive usual care for heart failure from the participating institutions. Subjects from both groups are instructed not to participate in group exercise classes or education offerings by the exercise facility.

### Procedures for intervention fidelity

Procedures to evaluate intervention fidelity are incorporated into the design of the study. The intervention fidelity plan addresses the training of those interacting with the participants, the delivery of the intervention, and the receipt and enactment of the intervention for the participants. Training sessions focused on consistency between sites and personnel. Standardized training manuals and scripts were used for training sessions of study personnel prior to the enrollment of participants. Booster training sessions occur every 6 months for data collectors and group session leaders and every 8–12 weeks for exercise coaches during the study. Delivery of the intervention by exercise coaches is scripted and actual delivery of the intervention components and length of the session are recorded each week in a database. This database is audited for adherence to the intervention and feedback is given to coaches at their meetings. Coaching sessions are video-recorded every few months to assess adherence to delivery of the intervention according to protocol. Group education session content is standardized with a pre-recorded video presentation followed by group discussion. Group session leaders fill out a self-report each session to document the discussion topics that were covered. Receipt and enactment of the intervention by participants in the HEART Camp group is gauged with exercise diaries, documentation from coaches, qualitative interviews, input and engagement surveys, receipt of intervention tools, and adherence to the exercise.

### Study outcomes and data collection

Table 3 describes data collection time points as well as outcome variables and measures specified by research aim. All questionnaire data is collected by interview according to standardized scripts and entered directly into an electronic database (REDCap) by the blinded research assistant [55]. The primary outcome of adherence is obtained through self-reported exercise diaries and validated by heart rate monitor (Polar Electro Inc., Lake Success, NY) data. All subjects are instructed to complete the diary and wear the heart rate monitor for each exercise session. Exercise coaches collect diaries and data from HC intervention participants during weekly coaching sessions and research personnel collect this data for the SC subjects by appointment every two months. Subjects are contacted by phone and diary information is collected by scripted interview if sessions or appointments are missed. Research assistants blinded to group assignment collect data on measures of physical function, psychological function, symptoms and quality of life (6 MWT, PROMIS-29, Dyspnea/Fatigue Scale and KCCQ), as well as measures related to the intervention mechanisms (knowledge, attitude, self-efficacy, self-management, and social support) at 6, 12 and 18 months (see Table 2).

**Table 3:** **Outcome measures and data collection time points by research aim**

Variable	Measure description (data collection time points)
	**Baseline & screening**
**Demographic and clinical variables Tool**	Demographic (e.g., race, gender, age, marital status)
	Clinical data (e.g., ejection fraction or EF, medications, BMI, comorbidities)
**Cardiopulmonary stress test**	Determine suitability for exercise (no significant ischemia or arrhythmias)
	Heart rate ranges and appropriate levels of exercise training
	**Primary outcome – Aim 1 (Baseline, 6, 12 and 18 months)**
**Adherence**	
**Self-report**	**Exercise diary** for self-report of sessions per week and minutes per session [56]
**Heart rate monitor**	**Heart rate monitor** (used as validation of self-reported exercise each week
	**Evaluation of intervention mechanisms – Aim 2 (Baseline, 6, 12 and 18 months)**
**Knowledge**	**Physical activity & heart disease I.Q.** – developed by NHLBI [57] 12 true/false items testing knowledge of how physical activity affects the heart
**Attitudes**	**Attitudes toward physical activity/exercise** - 8 items measure negative attitudes toward physical activity and 6 items measure positive attitudes toward physical activity [58]
**Self-efficacy**	**Barriers Self-Efficacy Scale (BARSE) –** 13 item scale measures self-efficacy or confidence in exercise behavior [59];
**Self-management**	**Physical Activity Self-Regulation Scale (PASR 12)**
	Self-monitoring (2 items), Goal setting (2 items), Eliciting social support (2 items), Reinforcements (2 items), Time management (2 items) and Relapse management (2 items) [60]
**Social support**	**Revenson Support Scale**
	Problematic support (4 items) and Positive support (16 items) [61]
	**Health outcomes – Aim 3 (Baseline, 6, 12 and 18 months)**
**Physical function**	**6 Minute Walk Test (6 MWT) –** Objective measure of functional capacity (sub-maximal). Distance walked in 6 minutes [62, 63]
**Psychological function**	**PROMIS-29 Profile v1.0:** Anxiety (4 item subscale), Depression (4 item subscale), Satisfaction with Social Role (4 item subscale) [64]
**Symptoms**	**Dyspnea/Fatigue Scale –** Measures the magnitude of the task that evokes dyspnea or fatigue, the magnitude of the pace at which the task is performed, and the associated functional impairment in general activities as a result of the symptoms [65]
**Quality of life**	**Kansas City Cardiomyopathy Questionnaire (KCCQ) –** 23-item disease-specific measure of quality of life in heart failure [66, 67]
	**Qualitative data – Aim 5**
**Open-Ended questions**	All subjects complete at **baseline**
**All subjects at baseline**	Survey prior experience with exercise, expectations related to exercise and outcomes from participation
**Open-ended comments on exercise diaries**	All subjects complete **each week** of the study
**All subjects each week**	What helped you to exercise this week? What made exercising a challenge this week? Other comments?
**One-on-one interviews**	Open-ended questions with probing (30 minutes)
**with HC subjects**	Interviews completed at **3, 6, 12 and 18** months
	Survey subjects’ perceptions of their experience with exercise adherence (e.g., challenges with adherence, helpful strategies, difficulties)
**One-on-one interviews with coaches**	Open-ended questions with probing (**every 6 months during active recruitment**)
	Questions about perceptions of exercise adherence experiences, strategies/difficulties working with patients, assessments of intervention components, descriptions of the intervention process for each phase
**One-on-one interviews with group session leaders**	Open-ended questions with probing (**every 6 months during active recruitment**)
	Questions about perceptions of exercise adherence experiences, strategies/difficulties working with patients, assessments of intervention components, descriptions of the intervention process

Qualitative data is obtained from all study participants at baseline testing with an open-ended questionnaire exploring perceptions of prior experiences with exercise, expectations related to exercise and outcome expectations from participation in the study (see Table 2). Additional qualitative data is collected from open-ended questions on the exercise diary of what helped with exercise and what made exercise a challenge during the week, as well as a general comment section. The baseline questionnaire and all comments from the diaries are transcribed and used to supplement the thematic analysis of the qualitative interview data. Participants in the HC intervention group complete qualitative interviews at 6, 12 and 18 months, as well as at 3 months to obtain participant perspectives during the adoption phase of exercise. Selected SC subjects (i.e., low adherers or high adherers) complete qualitative interviews at completion of the 18 month study period.

### Data analysis

Before proceeding with analysis, a careful descriptive study will be conducted to identify outliers and evaluate distributional assumptions of all variables. Primary analyses will be conducted consistent with the intent-to-treat paradigm, with each participant’s data analyzed according to group assignment. Patterns of missing data will be evaluated, and where assumptions are reasonable, analysis methods that accommodate partial cases such as mixed linear models or generalized estimating equations (GEE) will be used. For analyses requiring complete data, we will impute missing values using the EM algorithm or multiple imputation, as appropriate. To evaluate the central hypothesis, a one-tailed z-test will be used to compare the proportion of patients in each intervention group who are exercising on average ≥80% of the recommended 150 minutes per week at 18 months. A supplemental analysis will compare groups across the entire 18-month study period (testing main and interaction effects) on weekly minutes of activity at moderate or greater intensity and on specific health outcomes. Models of mediation and moderation of the effect of the intervention on adherence will be explored using regression methods.

## Discussion

This study will impact the management of HF by investigating the challenging problem of adherence to the HFSA guidelines for exercise in HF. Improving adherence to these evidence-based guidelines is expected to improve HF outcomes and reduce costs in this burdensome chronic illness. Furthermore, gaining a better understanding of what strategies are effective in promoting exercise adherence will help us to better design and implement future exercise trials in HF.

## Abbreviations

6 MWT: 6 minute walk test
ACEI: Angiotensin converting enzyme inhibitor
ANOVA: Analysis of variance
ARB: Angiotensin receptor blockers
BARSE: Barriers self-efficacy scale
BMI: Body mass index
CMS: Centers for medicare and medicaid services
CPX: Cardiopulmonary stress test
EF: Ejection fraction
GEE: Generalized estimating equations
HC: HEART camp intervention group
HEART: Heart failure exercise and resistance training
HF: Heart failure
HFpEF: Heart failure preserved ejection fraction
HFrEF: Heart failure reduced ejection fraction
HFSA: Heart failure society of America
HMC: NIH health maintenance consortium
HRR: Heart rate reserve
KCCQ: Kansas city cardiomyopathy questionnaire
MVO2: Mixed venous oxygen saturation
NHLBI: National heart, lung, and blood institute: national institute
NIH: National institutes of health
PI: Principal investigator
PROMIS-29: Patient reported outcome measurement information system profile instrument
RN: Registered nurse
RPE: Rating of perceived exertion
SC: Standard care.

## References

[1] Lindenfeld J, Albert NM, Boehmer JP, Collins SP, Ezekowitz JA, Givertz MM, Katz SD, Klapholz M, Moser DK, Rogers JG, Starling RC, Stevenson WG, Tang WH, Teerlink JR, Walsh MN, Heart Failure Society of America (2010). HFSA 2010 comprehensive heart failure practice guideline. J Card Fail.

[2] Evangelista LS, Kagawa-Singer M, Dracup K (2001). Gender differences in health perceptions and meaning in persons living with heart failure. Heart Lung.

[3] Gary R (2006). Exercise self-efficacy in older women with diastolic heart failure: results of a walking program and education intervention. J Gerontol Nurs.

[4] van der Wal MH, van Veldhuisen DJ, Veeger NJ, Rutten FH, Jaarsma T (2010). Compliance with non-pharmacological recommendations and outcome in heart failure patients. Eur Heart J.

[5] Piepoli MF, Conraads V, Corra U, Dickstein K, Francis DP, Jaarsma T, McMurray J, Pieske B, Piotrowicz E, Schmid JP, Anker SD, Solal AC, Filippatos GS, Hoes AW, Gielen S, Giannuzzi P, Ponikowski PP (2011). Exercise training in heart failure: from theory to practice. A consensus document of the heart failure association and the European association for cardiovascular prevention and rehabilitation. Eur J Heart Fail.

[6] Evangelista LS, Berg J, Dracup K (2001). Relationship between psychosocial variables and compliance in patients with heart failure. Heart Lung.

[7] Nieuwenhuis MM, Jaarsma T, van Veldhuisen DJ, Postmus D, van der Wal MH (2012). Long-term compliance with nonpharmacologic treatment of patients with heart failure. Am J Cardiol.

[8] Evangelista L, Doering LV, Dracup K, Westlake C, Hamilton M, Fonarow GC (2003). Compliance behaviors of elderly patients with advanced heart failure. J Cardiovasc Nurs.

[9] Conraads VM, Deaton C, Piotrowicz E, Santaularia N, Tierney S, Piepoli MF, Pieske B, Schmid JP, Dickstein K, Ponikowski PP, Jaarsma T (2012). Adherence of heart failure patients to exercise: barriers and possible solutions: a position statement of the study group on exercise training in heart failure of the heart failure association of the European society of cardiology. Eur J Heart Fail.

[10] van der Wal MH, Jaarsma T, Moser DK, Veeger NJ, van Gilst WH, van Veldhuisen DJ (2006). Compliance in heart failure patients: the importance of knowledge and beliefs. Eur Heart J.

[11] Jaarsma T, Abu-Saad HH, Dracup K, Halfens R (2000). Self-care behaviour of patients with heart failure. Scand J Caring Sci.

[12] Pihl E, Fridlund B, Martensson J (2011). Patients’ experiences of physical limitations in daily life activities when suffering from chronic heart failure; a phenomenographic analysis. Scand J Caring Sci.

[13] Rodriguez KL, Appelt CJ, Switzer GE, Sonel AF, Arnold RM (2008). “They diagnosed bad heart”: a qualitative exploration of patients’ knowledge about and experiences with heart failure. Heart Lung.

[14] Guiraud T, Granger R, Gremeaux V, Bousquet M, Richard L, Soukarié L, Babin T, Labrunée M, Sanguignol F, Bosquet L, Golay A, Pathak A (2012). Telephone support oriented by accelerometric measurements enhances adherence to physical activity recommendations in noncompliant patients after a cardiac rehabilitation program. Arch Phys Med Rehabil.

[15] Dolansky MA, Stepanczuk B, Charvat JM, Moore SM (2010). Women’s and men’s exercise adherence after a cardiac event. Does age make a difference?. Res Gerontol Nurs.

[16] Hambrecht R, Gielen S, Linke A, Fiehn E, Yu J, Walther C, Schoene N, Schuler G (2000). Effects of exercise training on left ventricular function and peripheral resistance in patients with chronic heart failure: a randomized trial. JAMA.

[17] McKelvie RS, Teo KK, Roberts R, McCartney N, Humen D, Montague T, Hendrican K, Yusuf S (2002). Effects of exercise training in patients with heart failure: the exercise rehabilitation trial (EXERT). Am Heart J.

[18] Keteyian SJ, Leifer ES, Houston-Miller N, Kraus WE, Brawner CA, O’Connor CM, Whellan DJ, Cooper LS, Fleg JL, Kitzman DW, Cohen-Solal A, Blumenthal JA, Rendall DS, Pina IL, HF-ACTION Investigators (2012). Relation between volume of exercise and clinical outcomes in patients with heart failure. J Am Coll Cardiol.

[19] Evangelista LS, Hamilton MA, Fonarow GC, Dracup K (2010). Is exercise adherence associated with clinical outcomes in patients with advanced heart failure?. Phys Sportsmed.

[20] Keteyian SJ (2010). Exercise in the management of patients with chronic heart failure. Curr Heart Fail Rep.

[21] Smart N (2010). Exercise training for heart failure patients with and without systolic dysfunction: an evidence-based analysis of how patients benefit. Cardiol Res Pract.

[22] Creswell JW, Fetters MD, Plano Clark VL, Morales A, Andrew S, Halcomb L (2009). Mixed methods intervention trials. Mixed Methods Research for Nursing and the Health Sciences.

[23] Creswell JW, Plano Clark VL (2011). Designing and Conducting Mixed Methods Research.

[24] Song M, Sandelowski M, Happ MB, Tashakkori A, Teddlie C (2010). Current practices and emerging trends in conducting mixed methods intervention studies in the health sciences. Handbook of Mixed Methods in Social & Behavioral Research.

[25] American College of Sports Medicine (2010). ACSM’s Guidelines for Exercise Testing and Prescription.

[26] Mandic S, Tymchak W, Kim D, Daub B, Quinney HA, Taylor D, Al-Kurtass S, Haykowsky MJ (2009). Effects of aerobic or aerobic and resistance training on cardiorespiratory and skeletal muscle function in heart failure: a randomized controlled pilot trial. Clin Rehabil.

[27] Pozehl B, Duncan K, Hertzog M, Norman JF (2010). Heart failure exercise and training camp: effects of a multicomponent exercise training intervention in patients with heart failure. Heart Lung.

[28] Whellan DJ, O’Connor CM, Pina I (2004). Training trials in heart failure: time to exercise restraint?. Am Heart J.

[29] Eakin EG, Bull SS, Riley KM, Reeves MM, McLaughlin P, Gutierrez S (2007). Resources for health: a primary-care-based diet and physical activity intervention targeting urban Latinos with multiple chronic conditions. Health Psychol.

[30] Albright CL, Pruitt L, Castro C, Gonzalez A, Woo S, King AC (2005). Modifying physical activity in a multiethnic sample of low-income women: one-year results from the IMPACT (increasing motivation for physical ACTivity) project. Ann Behav Med.

[31] Kumanyika SK, Shults J, Fassbender J, Whitt MC, Brake V, Kallan MJ, Iqbal N, Bowman MA (2005). Outpatient weight management in African-Americans: the healthy eating and lifestyle program (HELP) study. Prev Med.

[32] Ferrier S, Blanchard CM, Vallis M, Giacomantonio N (2011). Behavioural interventions to increase the physical activity of cardiac patients: a review. Eur J Cardiovasc Prev Rehabil.

[33] Williams SL, French DP (2011). What are the most effective intervention techniques for changing physical activity self-efficacy and physical activity behaviour–and are they the same?. Health Educ Res.

[34] Artinian NT, Fletcher GF, Mozaffarian D, Kris-Etherton P, Van Horn L, Lichtenstein AH, Kumanyika S, Kraus WE, Fleg JL, Redeker NS, Meininger JC, Banks J, Stuart-Shor EM, Fletcher BJ, Miller TD, Hughes S, Braun LT, Kopin LA, Berra K, Hayman LL, Ewing LJ, Ades PA, Durstine JL, Houston-Miller N, Burke LE, American Heart Association Prevention Committee of the Council on Cardiovascular Nursing (2010). Interventions to promote physical activity and dietary lifestyle changes for cardiovascular risk factor reduction in adults: a scientific statement from the American Heart Association. Circulation.

[35] Perry CK, Rosenfeld AG, Bennett JA, Potempa K (2007). Heart-to-heart: promoting walking in rural women through motivational interviewing and group support. J Cardiovasc Nurs.

[36] Yancey AK, McCarthy WJ, Harrison GG, Wong WK, Siegel JM, Leslie J (2006). Challenges in improving fitness: results of a community-based, randomized, controlled lifestyle change intervention. J Womens Health (Larchmt).

[37] Carels RA, Darby LA, Cacciapaglia HM, Douglass OM (2004). Reducing cardiovascular risk factors in postmenopausal women through a lifestyle change intervention. J Womens Health (Larchmt).

[38] Maeda U, Shen BJ, Schwarz ER, Farrell KA, Mallon S (2013). Self-efficacy mediates the associations of social support and depression with treatment adherence in heart failure patients. Int J Behav Med.

[39] Appel LJ, Champagne CM, Harsha DW, Cooper LS, Obarzanek E, Elmer PJ, Stevens VJ, Vollmer WM, Lin PH, Svetkey LP, Stedman SW, Young DR, Writing Group of the PREMIER Collaborative Research Group (2003). Effects of comprehensive lifestyle modification on blood pressure control: main results of the PREMIER clinical trial. JAMA.

[40] Toobert DJ, Strycker LA, Glasgow RE, Barrera M, Angell K (2005). Effects of the mediterranean lifestyle program on multiple risk behaviors and psychosocial outcomes among women at risk for heart disease. Ann Behav Med.

[41] Marcus BH, Napolitano MA, King AC, Lewis BA, Whiteley JA, Albrecht A, Parisi A, Bock B, Pinto B, Sciamanna C, Jakicic J, Papandonatos GD (2007). Telephone versus print delivery of an individualized motivationally tailored physical activity intervention: project STRIDE. Health Psychol.

[42] Marcus BH, Bock BC, Pinto BM, Forsyth LH, Roberts MB, Traficante RM (1998). Efficacy of an individualized, motivationally-tailored physical activity intervention. Ann Behav Med.

[43] Yehle KS, Plake KS (2010). Self-efficacy and educational interventions in heart failure: a review of the literature. J Cardiovasc Nurs.

[44] Jeffery RW, Wing RR, Thorson C, Burton LR (1998). Use of personal trainers and financial incentives to increase exercise in a behavioral weight-loss program. J Consult Clin Psychol.

[45] Green BB, McAfee T, Hindmarsh M, Madsen L, Caplow M, Buist D (2002). Effectiveness of telephone support in increasing physical activity levels in primary care patients. Am J Prev Med.

[46] Jacobs AD, Ammerman AS, Ennett ST, Campbell MK, Tawney KW, Aytur SA, Marshall SW, Will JC, Rosamond WD (2004). Effects of a tailored follow-up intervention on health behaviors, beliefs, and attitudes. J Womens Health (Larchmt).

[47] Conn VS, Hafdahl AR, Mehr DR (2011). Interventions to increase physical activity among healthy adults: meta-analysis of outcomes. Am J Public Health.

[48] Miller RB, Brickman SJ (2004). A model of future-oriented motivation and self-regulation. Educ Psychol Rev.

[49] Nilsen WJ, Haverkos L, Nebeling L, Taylor MV (2010). Maintenance of long-term behavior change. Am J Health Behav.

[50] Ory MG, Lee Smith M, Mier N, Wernicke MM (2010). The science of sustaining health behavior change: the health maintenance consortium. Am J Health Behav.

[51] Pozehl B, Duncan K, Norman J, Hertzog M, Walker A (2009). Daily physical activity levels as measured by self-report and accelerometry in patients with heart failure. J Card Fail.

[52] Conn VS, Hafdahl AR, Moore SM, Nielsen PJ, Brown LM (2009). Meta-analysis of interventions to increase physical activity among cardiac subjects. Int J Cardiol.

[53] Choitz P, Johnson MP, Berhane Z, Lefever G, Anderson JK, Eiser AR (2010). Urban fitness centers: removing barriers to promote exercise in underserved communities. J Health Care Poor Underserved.

[54] Richter DL, Wilcox S, Greaney ML, Henderson KA, Ainsworth BE (2002). Environmental, policy, and cultural factors related to physical activity in African American women. Women Health.

[55] Harris PA, Taylor R, Thielke R, Payne J, Gonzalez N, Conde JG (2009). Research electronic data capture (REDCap)–a metadata-driven methodology and workflow process for providing translational research informatics support. J Biomed Inform.

[56] Hertzog MA, Nieveen JL, Zimmerman LM, Barnason SA, Schulz PM, Miller CL, Rasmussen DA: **Longitudinal comparison of the RT3 and an activity diary with cardiac patients.** *J Nurs Meas* In press 10.1891/106137407782156363 18020168

[57] National Heart, Lung, and Blood Institute (NHLBI) (1996). Check your physical activity and heart disease IQ. [NIH Publication No. 96-3795].

[58] Nelson TD, Benson ER, Jensen CD (2010). Negative attitudes toward physical activity: measurement and role in predicting physical activity levels among preadolescents. J Pediatr Psychol.

[59] McAuley E, Mailey EL, Mullen SP, Szabo AN, Wojcicki TR, White SM, Gothe N, Olson EA, Kramer AF (2011). Growth trajectories of exercise self-efficacy in older adults: influence of measures and initial status. Health Psychol.

[60] Umstattd MR, Motl R, Wilcox S, Saunders R, Watford M (2009). Measuring physical activity self-regulation strategies in older adults. J Phys Act Health.

[61] Revenson TA, Schiaffino KM, Majerovitz SD, Gibofsky A (1991). Social support as a double-edged sword: the relation of positive and problematic support to depression among rheumatoid arthritis patients. Soc Sci Med.

[62] Cahalin LP, Mathier MA, Semigran MJ, Dec GW, DiSalvo TG (1996). The six-minute walk test predicts peak oxygen uptake and survival in patients with advanced heart failure. Chest.

[63] American Thoracic Society (2002). ATS statement: guidelines for the six-minute walk test. Am J Respir Crit Care Med March.

[64] Cella D, Riley W, Stone A, Rothrock N, Reeve B, Yount S, Amtmann D, Bode R, Buysse D, Choi S, Cook K, DeVellis R, DeWalt D, Fries JF, Gershon R, Hahn EA, Lai J, Gershon R, Hahn EA, Lai J, Pilkonis P, Revicki D, Rose M, Weinfurt K, Hays R (2010). Initial adult health item banks and first wave testing of the patient-reported outcomes measurement information system (PROMIS™) network: 2005–2008. J Clin Epidemiol Nov.

[65] Feinstein AR, Fisher MB, Pigeon JG (1989). Changes in dyspnea-fatigue ratings as indicators of quality of life in the treatment of congestive heart failure. Am J Cardiol.

[66] Green CP, Porter CB, Bresnahan DR, Spertus JA (2000). Development and evaluation of the Kansas city cardiomyopathy questionnaire: a new health status measure for heart failure. J Am Coll Cardiol.

[67] Spertus J, Peterson E, Conard MW, Heidenreich PA, Krumholz HM, Jones P, McCullough PA, Pina I, Tooley J, Weintraub WS, Rumsfeld JS, Cardiovascular Outcomes Research Consortium (2005). Monitoring clinical changes in patients with heart failure: a comparison of methods. Am Heart J.

[CR68] The pre-publication history for this paper can be accessed here: http://www.biomedcentral.com/1471-2261/14/172/prepub

